# Design, construction, and evaluation of the *BeneFit* socket: An adjustable temporary socket for a transradial prosthesis

**DOI:** 10.1097/PXR.0000000000000379

**Published:** 2024-11-27

**Authors:** Benedikt Baumgartner, Ivan Vujaklija, Oskar C. Aszmann, Anna Boesendorfer, Eugenijus Kaniusas, Agnes Sturma

**Affiliations:** 1 Clinical Laboratory for Bionic Extremity Reconstruction, Department of Plastic, Reconstructive and Aesthetic Surgery, Medical University of Vienna, Vienna, Austria; 2 Department Electrical Engineering and Automation, Aalto University, Helsinki, Finland; 3 Department of Plastic, Reconstructive and Aesthetic Surgery, Medical University of Vienna, Vienna, Austria; 4 Institute of Biomedical Electronics, Vienna University of Technology, Vienna, Austria; 5 Degree Program Physiotherapy, Department of Health Sciences, FH Campus Vienna University of Applied Sciences, Vienna, Austria

**Keywords:** early prosthetic fitting, amputation, upper extremity, prosthesis design, prosthesis adjustment, upper limb prosthesis

## Abstract

**Introduction:**

Upper limb prosthetics aim to restore function and aesthetics of a lost arm and/or hand, which affects a growing population. A “Golden Period,” the first month after the amputation, has been identified as the optimal time when a prosthesis should be fitted to amputees to maximize their chances for a fast and successful return to their daily life. This time window is often surpassed because of delays in approvals by health insurance companies and time requirements for producing personally customized and costly sockets. This study aims to develop an adaptable and versatile temporary transradial socket design capable of multidimensional adjustments and easy user fitting to better capitalize on the “Golden Period” and allow for early prosthetic training. In addition, the socket should be useable within research in need for flexible prosthesis as well as a hybrid prosthesis within the context of bionic reconstruction.

**Methods and Material:**

The socket was designed, 3D printed, and assembled as a wearable prototype, hosting all relevant myoelectric hand components. The estimated satisfaction with the socket was evaluated in a monocentric, explorative study with both, prosthetic users and experts in the field.

**Results and Discussion:**

The proposed design is able to change its diameter and length. Moreover, according to the conducted survey, it is perceived as satisfactory with respect to both user needs and expectations of the experts. “Weight,” “ease in donning and doffing,” and “breathability” were rated particularly well, whereas an improved, more versatile, and smaller design is needed for broad clinical use.

## Introduction

The loss of a hand is a traumatic experience that can decrease the autonomy of a person and impair their daily life.^1^ Moreover, the social and mental status of the affected individual are also often significantly impacted.^2^ The number of people suffering a limb loss is surprisingly high. In 2005, for example, 1.6 million people were living with an absent limb in the United States alone, and this number is projected to double by the year 2050.^3^ Out of these, roughly one-third suffer from upper limb amputations.^4^

A prosthetic device can help to cope with the loss as well as aid amputees in their daily life. Nevertheless, the rejection rates of upper limb prosthetics are staggering with a large review indicating that 1 in every 5 amputees abandons their prosthesis and a recent study indicating this number is as high as 44%.^5,6^ The most common reasons for the abandonment seem to be the comfort, the weight, and the limited practical function of the device itself.^6–9^ While the latter 2 are mainly dependent on the design of, sometimes rather complex, mechatronic system that is fitted to the user, the comfort of a prosthesis is highly dependent on its socket. These attachment interfaces should not be regarded as a low-level technology,^10–14^ although whose main design principles have been set back in 1960s and 1970s.^15^ Yet still, how well a socket fits has strong implications on the amount of time a user wears their prosthesis.^10^

A good socket needs to be easy to don and doff, and it needs to provide a stable base to mount a terminal device. Furthermore, a socket should be comfortable and able to reliably transfer the movement of the residual limb to the prosthesis.^16,17^ For the sake of hygiene, a prosthetic socket needs to be easy to disinfect, or better even washable. In addition, a socket should be biocompatible, lightweight, and restrict the movement as little as possible.

For the myoelectric prostheses, which rely on electromyographic signals from the underlying muscles of the residual limb to operate,^18–20^ a good socket fit is paramount for the sake of good function. Namely, as sockets host the necessary electromyographic electrodes, a constant and firm contact between the sensors and the skin is necessary for robust operation. The reliability of this interface is further influenced by noise-inducing factors such as sweat or the imposed terminal device load.^19,21^ Therefore, a socket should be breathable and firmly fitting. A recent survey-based study highlighted the need for lightweight, breathable, and adaptable socket solutions.^22^

Furthermore, the acceptance of a prosthesis is highly influenced by how early the fitting takes place as the timeframe between the amputation and the supply of a prosthetic device is crucial.^23^ It has been identified that the optimal period for patient fitting spans from 1^23^ to 6^24^ months. The effects of such early fits are predominantly observed in earlier return to work and the highly improved overall acceptance rates. For instance, Malone and colleagues^23^ found in their study that all of the patients who were injured at work and were fitted with a prosthesis during the first month, the so-called “Golden Period,” have successfully returned to work. However, only 15% of patients that were fitted later than that accomplished this. Meanwhile, research by Biddiss and colleagues^24^ showed that patients who were fitted with a prosthesis within the 6 months of their amputation were 16 times more likely to accept and consistently use their devices.

Unfortunately, in most healthcare systems, the “Golden Period” is currently surpassed. Reasons for this inefficiency can be many including administrative hurdles, specific medical circumstances, or the sheer complexity of many steps necessary in conventional socket fabrication.^25^ Furthermore, the residual limb often changes its shape in the first weeks after the amputation, making the early fabrication of a tailored socket difficult and uneconomic.^26^ Finally, the process of customizing an upper limb prosthetic device can be lengthy and tedious, hindered, among other obstacles, by the difficult and expensive process of custom socket manufacturing.

To better utilize this critical period right after the amputation, an adaptable temporary socket could be leveraged. This socket would easily conform to various patients' and their still changing residual limb geometries. Ideally, this adaptability would be self-contained, eliminating the need for frequent remanufacturing. In addition, the socket would facilitate quick and secure fastening, while offering flexibility for different sensor placements and attachment of various terminal devices.

Such an adaptable socket could also prove useful within the context of “hybrid hand fitting” where commonly a splint like construction hosting a prosthetic hand is mounted in parallel to patient’s own asensate hand that cannot be actively moved.^27^ The purpose of it is to evaluate patient’s capacity to control a prostheses, and in case of a good outlook, advantageous^28,29^ yet complex procedures such as bionic reconstruction could potentially be pursued.^30^ As this procedure involves an elective amputation, this temporary fitting and evaluation is essential in the decision-making process, and an access to an easily adjustable temporary socket attachment would be invaluable to the clinicians and patients.

Another application for an adjustable socket design lies within research itself, especially in evaluating novel control strategies in an experimental setting. The evaluation of these control strategies with actual patients and, therefore, prosthetic sockets is crucial, which can be difficult and expensive since the patient’s socket needs to be individually designed and constructed.^14^ In this context, the use of an adjustable socket, which can also be used as a hybrid prosthesis for able-bodied individuals, could prove highly beneficial. Analog to Hallworth et al,^14^ who designed a modular adjustable transhumeral prosthetic socket for evaluating myoelectric control, an adjustable socket for transradial patients could be of use.

Current customizable transradial solutions include the sleeves by Koalaa,^31^ which can adapt the diameter. Yet is only a passive device. Thus, to our knowledge, there is currently no temporary adjustable transradial prosthetic socket available for myoelectric devices. We, therefore, aimed to design, construct, and evaluate a first iteration of an adjustable, temporary, transradial socket, which can fit different patients as well as function as part of a hybrid prosthesis.

## Materials and methods

After initial consultation with clinicians and engineers focused on upper limb prosthetic rehabilitation, the initial features for a desirable adjustable socket were identified: The socket should be adjustable in length and diameter, easy to don and doff, provide stable base/reliable transfer movement of stump, be comfortable, biocompatible, lightweight, breathable, have adaptable electrode positioning, and a good range of motion. Different solutions on how to satisfy these basic requirements in a first prototype were iteratively discussed in several interdisciplinary team meetings, where the most promising avenue was then selected (see Supplemental Digital Content 3, http://links.lww.com/POI/A262 for more detail). This predominantly human-centered design approach, which leverages the knowledge and experience of diverse stakeholders in the field, may not include the systematic analysis typically used in more advanced product development stages. However, because of the complexity of the features required for such an adjustable socket system and the lack of previous attempts to achieve this, the emphasis was placed on flexibility, iteration, and responsiveness in the design process.

**Figure 1.**
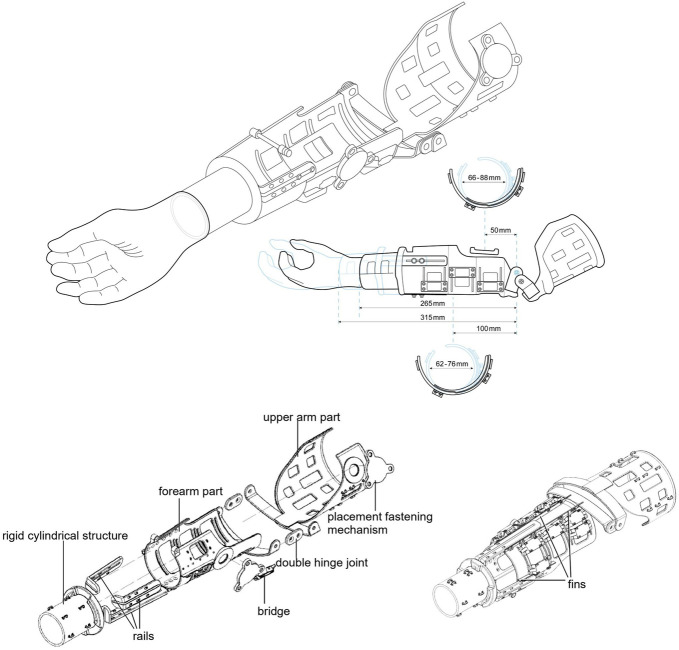
Above: Schematic drawing of BeneFit socket and attached end device: changeable dimensions visible; bottom left: isometric exploded view form above of hard shell; bottom right: isometric view form below; the upper arm part and forearm part are connected by a double hinge joint; multiple bridges mounted on forearm part; rails connect the adjustable forearm part and the rigid cylindrical structure at the distal end, form the transition from the adaptable structures to the rigid cylindrical structure.

The *BeneFit* socket (3D files freely available at https://github.com/BeneFit95/BeneFit) was designed using Autodesk Fusion 360 (Autodesk Inc., California). To provide the socket with a solid structure, which is able to reliably transfer the force, while simultaneously establishing a comfortable fit, the socket has 2 layers, a hard outer shell (see Figure 1) and a soft inner cushioning layer. To increase the comfort and minimize the injury risk, there are no sharp edges on the hard shell and the inner edges of the socket were bevelled. The cushioning material is placed on the inside of the hard shell to provide more comfort, since the direct contact of the hard shell with the stump could be painful.

### Socket design

To add the main function of adjustability to fit different patients, a compromise between changeability and stability had to be found. The socket has a modular design to be adaptable. To provide a secure enough fit, it has 2 main parts—an upper arm and a forearm part. They are connected by a double-hinge joint so that the prosthesis itself does not rely on a fixed center of rotation but adjusts to the rotation axis of the elbow of each patient, thereby offering a wider range of motion. The diameter can be adjusted by the easy-to-use fastening systems (RevoFit2^TM^ systems) with 1 hand, which should allow for easy donning and doffing.

At the distal end of the forearm, an adjusting screw establishes an adaptable but rigid connection, which adds stability. Four fins on the forearm part add rigidity in longitudinal direction, while conserving more elasticity needed for adaptability in transverse direction. Through 3 rails, the *BeneFit* socket is adjustable in length. This rail system also represents the transition from the adaptable structures to the rigid cylindrical structure intended to host the electrical components needed for a myoelectric prosthesis. The 2 parts of the socket feature mostly hollow structures to reduce the weight of the socket and make it more breathable. In addition, these numerous openings on the forearm part offer a variety of options for electrode placement needed to control the myoelectric prosthesis. The electrodes can be fixated using custom “bridges” in various positions. There are multiple slits and loops on the outside of the prosthesis where additional components can be attached (e.g., battery pack).

### Socket materials and fabrication

3MESH spacer fabric (6020 black) by Müller Textile Group is used as a cushioning material. It was deemed to be a good balance between reduced thickness (3 mm) while still possessing good cushioning properties. It was also chosen because of its high permeability, a good pressure distribution, high-quality soft touch, high durability, and low weight.^32^ The textile meets the standard 100 by Oeko Tex (class 1); it is, therefore, biocompatible.

The different parts of the outer shell were constructed with the help of a 3D printer (Markforged Mark Two^TM^ 3D-Printer (Markforged, Massachusetts). Onyx^TM^ was chosen since the micro carbon fiber–filled nylon is known for its strength and toughness. In addition, the fiber orientation can be used to create a transverse isotropic material. Thereby, the elasticity can be conserved in the radial direction, while simultaneously establishing rigidity in the longitudinal direction, an important advantage over more common materials.

**Figure 2.**
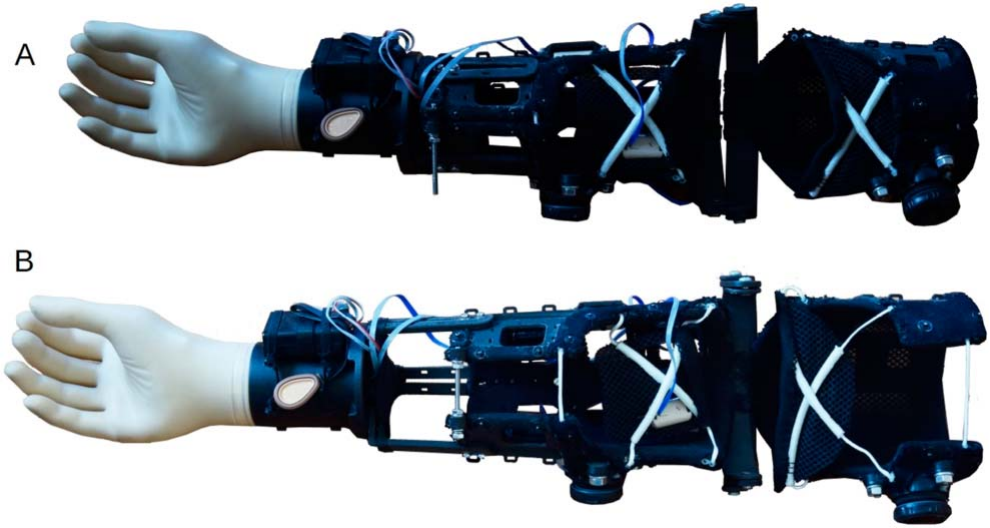
(a) Assembled prosthesis with minimal diameter and length; (b) assembled prosthesis with maximal diameter and length.

A layer height of 0.02 mm, a density of 33%, 2 full edge layers, 10 top/bottom layers, and triangle fill were set for all parts. After postprocessing, all different parts were assembled into a prototype (see Figure 2).

### Design evaluation

To have a fully functional prosthetic device, all necessary electric components, such as the MyoBock electrodes, electric wrist rotator 10S17, MyoRotronic, lamination ring, MyoEnergy Integral and SensorHand Speed (supplied by Ottobock Healthcare GmbH, Germany), were mounted on or within the socket.

The *BeneFit* socket, an experimental device not yet approved for clinical use, was evaluated in a monocentric, explorative study, which was approved by the ethic committee of the Medical University of Vienna (1724/2021). To get appropriate feedback from all the stakeholders, not only transradial amputees (n = 4) but experts on prosthetics from various professions (n = 9; occupational therapists [n = 2], a physiotherapist [n = 1], medical doctors [n = 2], orthopedic technicians [n = 3], textile engineer [n = 1]) were included in the survey. Before taking part in the study, the participants’ written informed consents were obtained.

The prototype was presented, the functions were explained, and participants had the chance to examine and mount it on a voluntary basis and see if they could control the terminal prosthesis. To assess if the socket could be used as a hybrid prosthesis for bionic reconstruction or within research itself, nonamputees could don it as well. A few choose to try a manipulate different objects. They could scrutinize it without any time constraint allowing them to test it until they felt comfortable to make a judgement. While there was not specific protocol to follow, most participants tried the prosthesis for about 10 minutes.

Afterward, they were presented with a questionnaire (which can be found in the Supplemental Digital Content 1, http://links.lww.com/POI/A260), with a few background questions slightly varying between users and experts. Based on the Quebec User Evaluation of Satisfaction with assistive Technology,^33^ it aimed to gauge participant’s estimated satisfaction with 10 different properties of the *BeneFit* socket. In addition, a short, semi-structured interview was conducted.

Since this an exploratory study of a first prototype, it aimed to gather broad feedback from different perspectives and used descriptive statistics to describe the results where applicable. To interpret the data, mean, median, most frequent value, maximum, minimum, standard deviation, variance, and the 25th and 75th percentile were calculated using SPSS 27 (IBM, Armonk, NY). Microsoft Office LTSC Professional Plus 2021 (Microsoft Corporation, Redmond, Washington) was used to create the boxplots for all the participants, the end users, and experts, respectively.

## Results

The *BeneFit* socket is biocompatible and provides an option to mount electrodes in different positions. The design is easy to adjust in diameter and length and can be considered light-weight. The socket itself weighs 500 g, and if a typical terminal device and other electronic components are included, the weight increases to about 1100 g, depending on the components. The forearm part can change its length from 265 to 315 mm. The diameter of the *BeneFit* socket can also be adjusted using the RevoFit2^TM^ system or the adjusting screw. The different diameters can be seen in Table 1. The different dimensions of the end user can be obtained from Table 2.

**Table 1.**
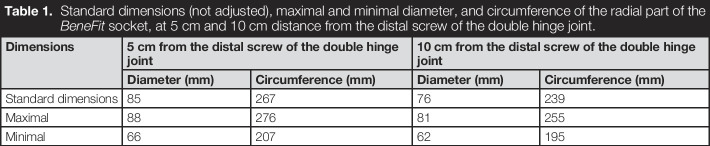
Standard dimensions (not adjusted), maximal and minimal diameter, and circumference of the radial part of the *BeneFit* socket, at 5 cm and 10 cm distance from the distal screw of the double hinge joint.

Dimensions	5 cm from the distal screw of the double hinge joint		10 cm from the distal screw of the double hinge joint	
	Diameter (mm)	Circumference (mm)	Diameter (mm)	Circumference (mm)
Standard dimensions	85	267	76	239
Maximal	88	276	81	255
Minimal	66	207	62	195

**Table 2.**
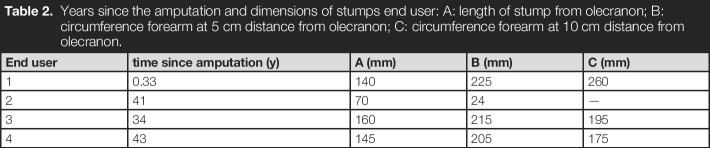
Years since the amputation and dimensions of stumps end user: A: length of stump from olecranon; B: circumference forearm at 5 cm distance from olecranon; C: circumference forearm at 10 cm distance from olecranon.

End user	time since amputation (y)	A (mm)	B (mm)	C (mm)
1	0.33	140	225	260
2	41	70	24	—
3	34	160	215	195
4	43	145	205	175

**Figure 3.**
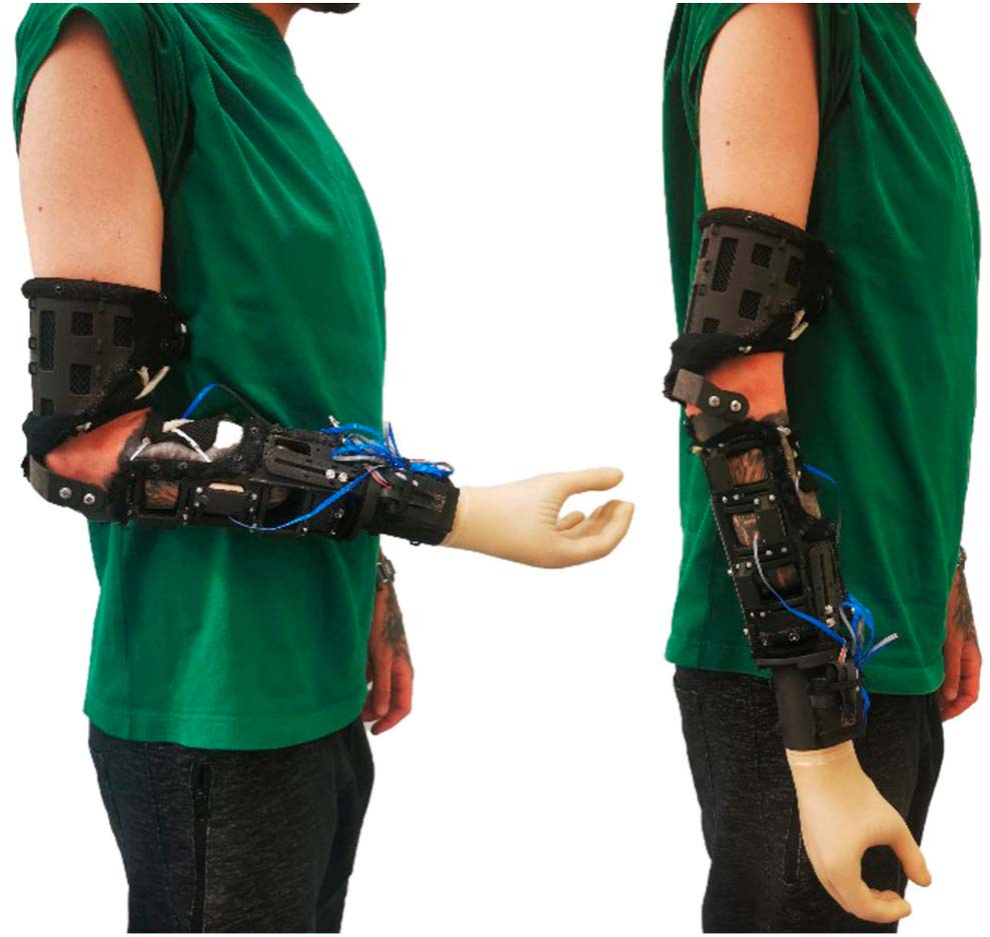
Volunteer/amputee wearing the *BeneFit* socket on the right side.

All end users were male, between 30 and 45 years of age. Two users had a body mass index within the normal range and the other 2 were at the lower end of the obese range (mean 23.63; SD = 2.56). All participants without an amputation, who attempted to don and doff the *BeneFit* socket, reported a relatively good fit and were able to address all the functions of the prosthetic hand. Three of the 4 amputees participating were able to don the socket and move their stump with the prosthesis attached to it as a whole (see Figure 3). One was not able to don the socket, because of the unconventional shape of his stump (almost mace shaped). However, all of the amputees would have preferred the option to reduce the diameter of the socket further to improve the fit. Because of the rather large dimensions, only 1 amputee tried to control the prosthetic hand and was successful in addressing the functions.

### Survey

**Figure 4.**
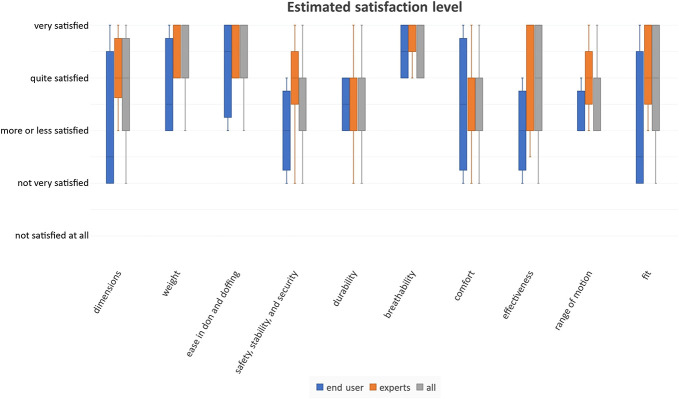
Boxplots of estimated satisfaction level of end users, experts, and combined.

**Figure 5.**
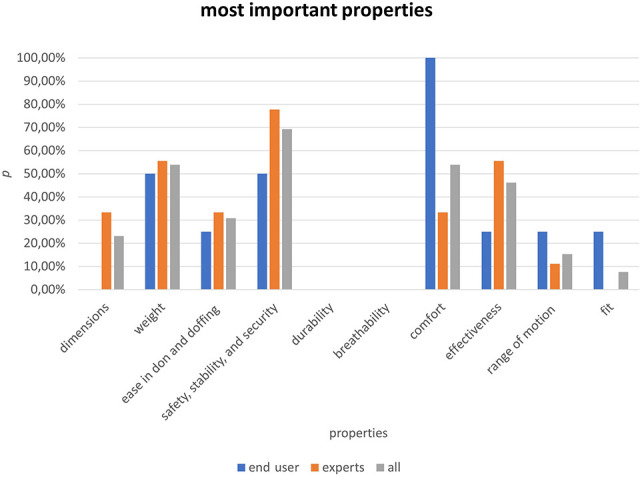
Bar chart: probability (*p*) of each property for being named as one of the 3 most important ones for end user, experts, and combined.

The results from the survey can be seen in Table 3 and Figure 4. The *BeneFit* socket is judged to be “quite satisfactory” if averaged over all the different properties. It scores especially well for the “ease in donning and doffing” and for the “breathability.” The group of experts tended to be more pleased by the design of the socket than the end users, especially in the categories “dimensions,” “weight,” “safety, stability and security,” “effectiveness,” “range of motion,” and “fit.” The perceived importance of each property of a prosthetic socket can be seen in Figure 5. The raw data from the questionnaires can be found in the Supplemental Digital Content 2, http://links.lww.com/POI/A261.

**Table 3.**
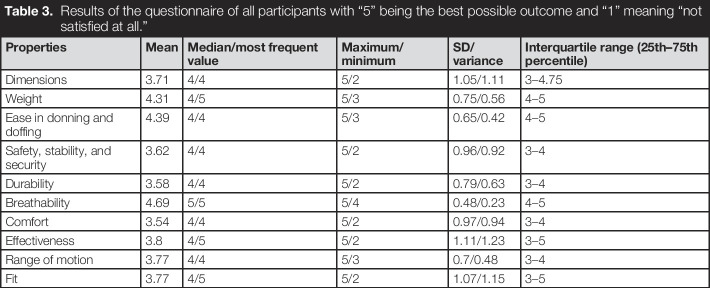
Results of the questionnaire of all participants with “5” being the best possible outcome and “1” meaning “not satisfied at all.”

Properties	Mean	Median/most frequent value	Maximum/minimum	SD/variance	Interquartile range (25th–75th percentile)
Dimensions	3.71	4/4	5/2	1.05/1.11	3–4.75
Weight	4.31	4/5	5/3	0.75/0.56	4–5
Ease in donning and doffing	4.39	4/4	5/3	0.65/0.42	4–5
Safety, stability, and security	3.62	4/4	5/2	0.96/0.92	3–4
Durability	3.58	4/4	5/2	0.79/0.63	3–4
Breathability	4.69	5/5	5/4	0.48/0.23	4–5
Comfort	3.54	4/4	5/2	0.97/0.94	3–4
Effectiveness	3.8	4/5	5/2	1.11/1.23	3–5
Range of motion	3.77	4/4	5/3	0.7/0.48	3–4
Fit	3.77	4/5	5/2	1.07/1.15	3–5

On average, all participants of the study supported the idea and concept of the *BeneFit* socket. They could imagine using this or a similar prosthesis as an early fitting in the clinical setting for training and testing purposes. For long-term use in daily life, they favored a conventional socket.

### Discussion

Providing amputees a prosthesis within the first months after amputation could not only improve the acceptance rate of prosthetic devices but also the time to return to work could be shortened.^23^ An adjustable prosthesis, which can change its shape to fit individual—and still changing—stumps, could provide patients the opportunity to start their prosthetic training as soon as possible. Furthermore, an adjustable socket of this kind could be beneficial for clinical studies, when new terminal devices or novel control strategies are being tested or for hybrid fittings within the context of bionic reconstruction.

For such a socket, the adaptability is the distinguishing feature. Nevertheless, it also needs to be lightweight, comfortable, and able to achieve a secure fit to the stump. In addition, it should be easy to don and doff as well as breathable, have a good range of motion, and mount electrodes in different individual positions. Our collected data indicate that the concept of the *BeneFit* socket is perceived to fulfil these requirements. The satisfaction with the weight of the socket (mean 4.31; SD = 0.75), ease of don/doffing the socket (mean 4.39; SD = 0.65), and breathability (mean 4.69; SD = 0.48) are the 3 highest scoring properties of the socket. Also, the *BeneFit* socket shows relatively good satisfaction in the categories of comfort (mean 3.54; SD = 0.97), range of motion (mean 3.77; SD = 0.7), safety, stability and security (mean 3.62; SD = 0.96), as well as fit (mean 3.77; SD = 1.07), but should be improved in a revised version.

Based on the conducted interviews and questionnaires, the concept behind the *BeneFit* socket was seen as a promising idea. There might, however, be a bias in this study, since the survey was done face to face with the creator of the *BeneFit* socket, the participant might be inclined to give more positive feedback. This can be explained the social desirability bias.^34^ Individuals probably tend to provide more positive responses because of the increased desirability of such answers, which will consequently enhance their likability in the interviewer’s perception.

The dimensions of the socket are better suited to individuals without amputations. When comparing Table 1 (minimal circumference) and Table 2 (circumference of amputees), some small overlap can be observed. More overlap is noticeable, if the minimal circumference of the BeneFit socket (195 mm at 100 mm distance from elbow crook) and the forearm circumference at 12 cm distance from the olecranon of persons without amputations (from 202 to 243 mm) are matched.^35^ This explains why the experts achieved a better fit than the amputees. Therefore, the *BeneFit* socket is currently better suited to be used as a hybrid socket for research, and not yet as a temporary socket for amputees. However, the concept behind the socket seems to be a viable option for amputees as well, especially if the dimension were decreased in a new model.

Continuing toward a revised design—the second generation of the *BeneFit* socket, will be scaled down in diameter and length to achieve a more secure and comfortable fit for amputees. Here, it is important to note that amputees may also suffer from severe nerve injuries, which add to the shrinkage of their residual limb, as was the case in 2 of the 4 included users.

The current prosthetic socket is disinfectable, although the hygienic aspects could be further improved by making the cushioning layer interchangeable with snap or similar fasteners.

In future iterations, different printing material as well as more design options for the different functions, such as for example, a different method to fixate electrodes, could be tried. Building on this essential initial design, which enabled us to gather preliminary stakeholder feedback, we can now incorporate Finite-Element-Methode analysis in the upcoming redesign process. This will help improve fit, comfort, and safety; optimize the structural design; and reduce the amount of material needed, thereby further reducing the overall weight. Moving forward, a structured evaluation with more participants, especially end users, will be pursued. The redesigned socket should be also assessed using functional tests, which should provide more comprehensive insight into the capabilities of the *BeneFit* socket.

## Conclusions

With a sufficiently versatile temporary socket that can be adjusted along different dimensions, it should be possible to fit different patients with just a few quick adaptations. This would allow for an early prosthetic training, which would potentially take full advantage of the “Golden Period.” After the first design, the construction and assembly of the adjustable, temporary, transradial *BeneFit* socket, a survey was conducted to evaluate the socket’s benefits and possible shortcomings with experts and end users. As 1 goal was to make the socket freely available for all, its building instructions and blueprints are available on GitHub. The socket is able to adapt its shape to fit different individuals. Because of its dimensions, the current model of the *BeneFit* socket seems more suited to be used as a research tool for abled-bodied people or a hybrid prosthesis than for amputees. Nevertheless, the concept could be promising as temporary solution after adjustments. The overall estimated satisfaction, averaged for all participants and all the categories, is 3.92/5.00 and, therefore, fairly close to the “quite satisfactory” grade. In addition, in the conducted survey, no participant described any of the socket’s features as being “not satisfactory at all.” Future iterations of the socket should aim to include a reduction feature or a version with an even smaller inner diameter, ensuring a good fit for thinner residual limbs found in some amputees. Before more pragmatic clinical translation of the next generation of the *BeneFit socket*, an in-dept evaluation of the system, ranging from finite element method analysis to structured functional testing with amputees, should be done as to understand the full potential of the solution.

## Funding

The project received funding from the European Research Council under the European Union’s Horizon 2020 research and innovation program (grant agreement No 810346). Müller Textile Group provided the 3MESH spacer fabric after an inquiry without receiving payment. Ottobock Healthcare GmbH provided the RevoFit2^TM^ system after an inquiry without receiving payment as well as loaned the parts needed for the myoelectric terminal device without any fees.

## Declaration of conflicting interest

The authors declare no conflict of interest.

## Supplemental material

Supplemental material for this article is available in this article. Direct URL citation appears in the text and is provided in the HTML and PDF versions of this article on the journal’s Web site (www.POIjournal.org).

## Supplementary Material

SUPPLEMENTARY MATERIAL
