# Supplementary material for: Design, construction, and evaluation of the BeneFit socket: An adjustable temporary socket for a transradial prosthesis
Source: Prosthet Orthot Int. 2024 Nov 27;49(5):515–22. doi: 10.1097/PXR.0000000000000379 (PMC12509435; doi:10.1097/PXR.0000000000000379)
Supplement: SUPPLEMENTARY MATERIAL [file poi-49-515-s001.docx]

| Participant-code: | |  | | | | | | | |
| --- | --- | --- | --- | --- | --- | --- | --- | --- | --- |
| Gender: | |  | | | | | | | |
| Affected Side: | |  | | | | | | | |
| Length from the crook of the elbow to the tip of the limb: | | | | | |  | | | |
| The circumference of the forearm (5 cm from the crook of the elbow ): | | | | | |  | | | |
| The circumference of the forearm (10 cm from the crook of the elbow): | | | | | |  | | | |
| Time passed since loss of upper limb: | | | | | |  | | | |
|  | | | | | | | | | |
| Please give your estimated satisfaction with the following properties of the prosthetic socket. Please circle or mark the one number that best describes your degree of satisfaction. Please do not leave any question unanswered: | | | | | | | | | |
| 1 | 2 | | 3 | | 4 | | | 5 | |
| not satisfied at all | not very satisfied | | more or less satisfied | | quite satisfied | | | very satisfied | |
| How satisfied are you with | | | | | | | | | |
| 1. the dimensions (size, height, length, width) of the prosthetic socket?  *Comments:* | | | | 1 | 2 | | 3 | 4 | 5 |
| 2. the weight of your assistive the prosthetic socket?  *Comments:* | | | | 1 | 2 | | 3 | 4 | 5 |
| 3. the ease in donning and doffing the prosthetic socket?  *Comments:* | | | | 1 | 2 | | 3 | 4 | 5 |
| 4. how safe, stable and secure the prosthetic socket is?  *Comments:* | | | | 1 | 2 | | 3 | 4 | 5 |
| 5. the durability (endurance, resistance to wear) of the prosthetic socket?  *Comments:* | | | | 1 | 2 | | 3 | 4 | 5 |
| 6. how breathable the prosthetic socket is?  *Comments:* | | | | 1 | 2 | | 3 | 4 | 5 |
| 7. how comfortable the prosthetic socket is?  *Comments:* | | | | 1 | 2 | | 3 | 4 | 5 |
| 8. how effective the prosthetic socket is (the degree to which your device meets your needs)?  *Comments:* | | | | 1 | 2 | | 3 | 4 | 5 |
| 9. the remaining range of motion of the prosthetic socket?  *Comments:* | | | | 1 | 2 | | 3 | 4 | 5 |
| 10. how well the prosthetic socket fits (adjustable in dimensions for you)  *Comments:* | | | | 1 | 2 | | 3 | 4 | 5 |

Below is the list of the same 10 properties of prosthetic sockets. PLEASE SELECT THE THREE PROPERTIES that you consider to be the most important to you. Please put an X in the 3 boxes of your choice.

| □ | 1. dimensions | □ | 6. permeable to air |
| --- | --- | --- | --- |
| □ | 2. weight | □ | 7. comfort |
| □ | 3. ease in donning and doffing | □ | 8. effectiveness |
| □ | 4. safety, stability and security | □ | 9. range of motion |
| □ | 5. durability | □ | 10. fit |

| General comments or suggestions (for example is there special feature/function of the prosthetic socket you would like). Additionally to the comments here, you can also elaborate on you points in the consecutive interview: |
| --- |
|  |

| Participant-code: | |  | | | | | | |
| --- | --- | --- | --- | --- | --- | --- | --- | --- |
| Gender: | |  | | | | | | |
| Occupation: | |  | | | | | | |
| Work experience/duration in the area: | | |  | | | | | |
|  | | | | | | | | |
| Please give your estimated satisfaction with the following properties of the prosthetic socket. Please circle or mark the one number that best describes your degree of satisfaction. Please do not leave any question unanswered: | | | | | | | | |
| 1 | 2 | | 3 | | 4 | | 5 | |
| not satisfied at all | not very satisfied | | more or less satisfied | | quite satisfied | | very satisfied | |
| How satisfied are you with | | | | | | | | |
| 1. the dimensions (size, height, length, width) of the prosthetic socket?  *Comments:* | | | | 1 | 2 | 3 | 4 | 5 |
| 2. the weight of your assistive the prosthetic socket?  *Comments:* | | | | 1 | 2 | 3 | 4 | 5 |
| 3. the ease in donning and doffing the prosthetic socket?  *Comments:* | | | | 1 | 2 | 3 | 4 | 5 |
| 4. how safe, stable and secure the prosthetic socket is?  *Comments:* | | | | 1 | 2 | 3 | 4 | 5 |
| 5. the durability (resistance to wear) of the prosthetic socket?  *Comments:* | | | | 1 | 2 | 3 | 4 | 5 |
| 6. how breathable the prosthetic socket is?  *Comments:* | | | | 1 | 2 | 3 | 4 | 5 |
| 7. how comfortable the prosthetic socket is?  *Comments:* | | | | 1 | 2 | 3 | 4 | 5 |
| 8. how effective the prosthetic socket is (the degree to which your device meets your needs)?  *Comments:* | | | | 1 | 2 | 3 | 4 | 5 |
| 9. the range of motion of the prosthetic socket?  *Comments:* | | | | 1 | 2 | 3 | 4 | 5 |
| 10. how well the prosthetic socket fits (adjustable in dimensions for you)  *Comments:* | | | | 1 | 2 | 3 | 4 | 5 |

Below is the list of the same 10 properties of prosthetic sockets. PLEASE SELECT THE THREE PROPERTIES that you consider to be the most important to you. Please put an X in the 3 boxes of your choice.

| □ | 1. dimensions | □ | 6. permeable to air |
| --- | --- | --- | --- |
| □ | 2. weight | □ | 7. comfort |
| □ | 3. ease in donning and doffing | □ | 8. effectiveness |
| □ | 4. safety, stability and security | □ | 9. range of motion |
| □ | 5. durability | □ | 10. fit |

| General comments or suggestions (for example is there special feature/function of the prosthetic socket you would like). Additionally to the comments here, you can also elaborate on you points in the consecutive interview: |
| --- |
|  |
