# Supplementary material for: Design, construction, and evaluation of the BeneFit socket: An adjustable temporary socket for a transradial prosthesis
Source: Prosthet Orthot Int. 2024 Nov 27;49(5):515–22. doi: 10.1097/PXR.0000000000000379 (PMC12509435; doi:10.1097/PXR.0000000000000379)
Supplement: SUPPLEMENTARY MATERIAL [file poi-49-515-s002.docx]

| Code | 01 | 02 | 03 | 04 |
| --- | --- | --- | --- | --- |
| Expert/User | expert | user | expert | expert |
| Gender | female | male | male | female |
| Time spent working in area/Time elapsed since amputation | 1,75 years | 0,33 years | 0,5 years | 10 years |
| Dimensions Stump:  Length form crook of the elbow circumference 5 cm from crook  circumference 10 cm from crook |  | 14 cm  22,5 cm  26 cm |  |  |
|  |  |  |  |  |
| How satisfied are you with (1-5): |  |  |  |  |
| 1. the dimensions (size, height, length, width) of the prosthetic socket?  *Comments:* | 5 | 5 | 4  *whole length for small persons too long* | 3  *probably to long, for middle- to long stumps, BOA buttons press against the body* |
| 2. the weight of your assistive the prosthetic socket?  *Comments:* | 4  *with prosthetic hand weight more towards distal end* | 5 | 4  *for small persons too heavy?* | 5  *very lightweight* |
| 3. the ease in donning and doffing the prosthetic socket?  *Comments:* | 4 | 5 | 5 | 4  *generally very easy, BOA needs some force to open* |
| 4. how safe, stable and secure the prosthetic socket is?  *Comments:* | 4 | 3 | 5 | 4  *but difficult to assess, since I am not amputated and the prosthetic hand is missing* |
| 5. the durability (endurance, resistance to wear) of the prosthetic socket?  *Comments:* | 4  *cleaning of cushioning material* | 3 | 5 | 3  *BOA buttons stand out far, easy to bump into something -> abrasion* |
| 6. how breathable the prosthetic socket is?  *Comments:* | 5 | 5 | 5 | 5 |
| 7. how comfortable the prosthetic socket is?  *Comments:* | 4 | 5 | 4  *pressure marks cords, edges?* | 3  *very lightweight, agility is good, but cords constrict -> nothing for long periods of time* |
| 8. how effective the prosthetic socket is (the degree to which your device meets your needs)?  *Comments:* | 5 | 3 | 5 | 5  *for the testing, if a prosthesis can be controlled, as a first contact for patient = excellent* |
| 9. the remaining range of motion of the prosthetic socket?  *Comments:* | 5 | 4 | 4  *see next* | 4  *through the constriction full flexion not possible* |
| 10. how well the prosthetic socket fits (adjustable in dimensions for you)  *Comments:* | 5 | 5 | 4  *elbow axis too short* | 4  *circumference good, length not estimable* |
| most important satisfaction items | dimensions  safety, stability and security  effectiveness | weight  ease in donning and doffing  comfort | dimensions  ease in donning and doffing  effectiveness | weight  safety, stability and security  comfort |
| General comments or suggestions | individual adjustment of the electrode position (simple) | - | rotational unit y/n for interims supply  durability for interims supply | rework BOA system: f.e. place it on the upper arm on the back, smaller BOA system  cords must not be on the skin, possible constriction of the blood flow hurts especially during the flexion of the arm  shorten the length = basis  -> distal probably no electrode position necessary |

| Code | 05 | 06 | 07 | 08 |
| --- | --- | --- | --- | --- |
| Expert/User | expert | expert | expert | user |
| Gender | male | male | female | male |
| Time spent working in area/Time elapsed since amputation | 24 years | 3 years | 1 year | 41 years |
| Dimensions Stump:  Length form crook of the elbow circumference 5 cm from crook  circumference 10 cm from crook |  |  |  | 7 cm  24 cm  - |
|  |  |  |  |  |
| How satisfied are you with (1-5): |  |  |  |  |
| 1. the dimensions (size, height, length, width) of the prosthetic socket?  *Comments:* | 4  *BOA system prox. very prominent* | 3,5 | 4 | 3  *very many parts stand away, edges…* |
| 2. the weight of your assistive the prosthetic socket?  *Comments:* | 4 | 4 | 5 | 3  *all components like the battery are very distal* |
| 3. the ease in donning and doffing the prosthetic socket?  *Comments:* | 5 | 4 | 4 | 3  *user can only be unilateral, hand has to function very well* |
| 4. how safe, stable and secure the prosthetic socket is?  *Comments:* | 4 | 3 | 4 | 3  *can only be used restricted during daily life, for rehabilitation okay* |
| 5. the durability (endurance, resistance to wear) of the prosthetic socket?  *Comments:* | -  *test period too short for judgement* | 2 | 4 | 4  *3D print with all advantages and disadvantages* |
| 6. how breathable the prosthetic socket is?  *Comments:* | 4 | 5 | 4 | 4  *yes, but only with additional textile liner, else might be too cold* |
| 7. how comfortable the prosthetic socket is?  *Comments:* | 4 | 2 | 3  *constricts* | 2  *openings to big and have to be covered* |
| 8. how effective the prosthetic socket is (the degree to which your device meets your needs)?  *Comments:* | 5 | 3 | 4 | 2  *comfort restricted since across the elbow* |
| 9. the remaining range of motion of the prosthetic socket?  *Comments:* | 5  *Impressive/ very good solution for elbow* | 3,5 | 3  *parts of the socket stand away* | 3  *could be problematic for very short stumps* |
| 10. how well the prosthetic socket fits (adjustable in dimensions for you)  *Comments:* | 5 | 3,5 | 4 | 2  *Circumference too big, skin gets pinched* |
| most important satisfaction items | dimensions  weight  effectiveness | weight  safety, stability and security  effectiveness | weight  safety, stability and security  comfort | weight  comfort  range of motion |
| General comments or suggestions | cushioning material should be wash- and changeable  cords of the BOA system constrict to some extent  length and width adaption are top  truly easy electrode positioning | Suggestion: flexible plastic pad + Velcro + cushion | - | - |

| Code | 09 | 10 | 11 | 12 |
| --- | --- | --- | --- | --- |
| Expert/User | user | expert | user | expert |
| Gender | male | male | male | male |
| Time spent working in area/Time elapsed since amputation | 11 years | 4 years | 11 years | 27 years |
| Dimensions Stump:  Length form crook of the elbow circumference 5 cm from crook  circumference 10 cm from crook | 16 cm  21,5 cm  19,5 cm |  | 14 cm  20,5 cm  17,5 cm |  |
|  |  |  |  |  |
| How satisfied are you with (1-5): |  |  |  |  |
| 1. the dimensions (size, height, length, width) of the prosthetic socket?  *Comments:* | 2  *too big* | 5 | 2 | 4 |
| 2. the weight of your assistive the prosthetic socket?  *Comments:* | 3 | 5 | 4 | 5 |
| 3. the ease in donning and doffing the prosthetic socket?  *Comments:* | 4 | 5 | 5 | 4 |
| 4. how safe, stable and secure the prosthetic socket is?  *Comments:* | 2 | 5 | 4 | 4 |
| 5. the durability (endurance, resistance to wear) of the prosthetic socket?  *Comments:* | 3 | 4 | 4  *within the scope of an interims supply* | 4 |
| 6. how breathable the prosthetic socket is?  *Comments:* | 4 | 5 | 5 | 5 |
| 7. how comfortable the prosthetic socket is?  *Comments:* | 3  *Upper arm good, forearm too loose* | 3 | 4 | 4 |
| 8. how effective the prosthetic socket is (the degree to which your device meets your needs)?  *Comments:* | 3 | 5 | 4 | 3 |
| 9. the remaining range of motion of the prosthetic socket?  *Comments:* | 3 | 4 | 3  *difficult to estimate because of the size* | 4 |
| 10. how well the prosthetic socket fits (adjustable in dimensions for you)  *Comments:* | 2 | 5 | 3  *see above* | 3 |
| most important satisfaction items | safety, stability and security  comfort  fit | ease in donning and doffing  safety, stability and security  comfort | safety, stability and security  comfort  effectiveness | weight  safety, stability and security  range of motion |
| General comments or suggestions | use smaller flatter BOA systems  place battery more proximal  reduce screw connections  offer different sizes  make the electrode positioning more flexible | pay attention to the cushioning of the cords  flexible  positioning of electrodes important | - | - |

| Code | 13 |  |  |  |
| --- | --- | --- | --- | --- |
| Expert/User | expert |  |  |  |
| Gender | male |  |  |  |
| Time spent working in area/Time elapsed since amputation | aprox. 40 years |  |  |  |
|  |  |  |  |  |
| How satisfied are you with (1-5): |  |  |  |  |
| 1. the dimensions (size, height, length, width) of the prosthetic socket?  *Comments:* | -  *too big* |  |  |  |
| 2. the weight of your assistive the prosthetic socket?  *Comments:* | 5 |  |  |  |
| 3. the ease in donning and doffing the prosthetic socket?  *Comments:* | 5 |  |  |  |
| 4. how safe, stable and secure the prosthetic socket is?  *Comments:* | 2 |  |  |  |
| 5. the durability (endurance, resistance to wear) of the prosthetic socket?  *Comments:* | 3 |  |  |  |
| 6. how breathable the prosthetic socket is?  *Comments:* | 5 |  |  |  |
| 7. how comfortable the prosthetic socket is?  *Comments:* | 5 |  |  |  |
| 8. how effective the prosthetic socket is (the degree to which your device meets your needs)?  *Comments:* | 2,5 |  |  |  |
| 9. the remaining range of motion of the prosthetic socket?  *Comments:* | 3,5 |  |  |  |
| 10. how well the prosthetic socket fits (adjustable in dimensions for you)  *Comments:* | 3,5 |  |  |  |
| most important satisfaction items | ease in donning and doffing  safety, stability and security  effectiveness |  |  |  |
| General comments or suggestions |  |  |  |  |
