# Supplementary material for: Design, construction, and evaluation of the BeneFit socket: An adjustable temporary socket for a transradial prosthesis
Source: Prosthet Orthot Int. 2024 Nov 27;49(5):515–22. doi: 10.1097/PXR.0000000000000379 (PMC12509435; doi:10.1097/PXR.0000000000000379)
Supplement: SUPPLEMENTARY MATERIAL [file poi-49-515-s003.docx]

| **Requirement** | **Feature** | **Chosen solution** | **Alternative solutions** | | **Reasons** |
| --- | --- | --- | --- | --- | --- |
| - adjustability  (length) | rails | 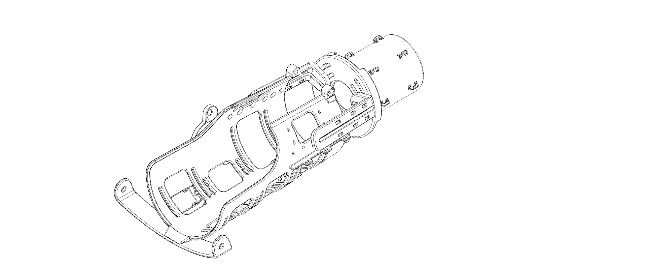L rails | 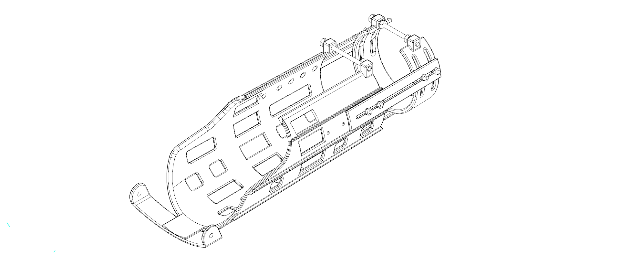I rails |  | - diameter socket independent of the  diameter of quick disconnect adapter |
| (diameter)  + stable base | general shape of cross section of the outside layer | 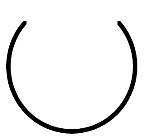open Circle | 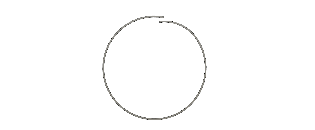snail | rails with Band around  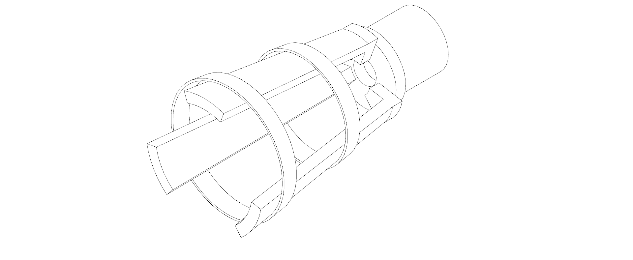 | - more comfort  - better transfer of movement from  stump to terminal device |
|  | material of 3D print | Onxy ^TM^ | common material PLA |  | - isotropic, more elasticity in radial  direction, while conserving stability  in longitudinal direction |
| (diameter)  + don/doff | fastening mechanism | 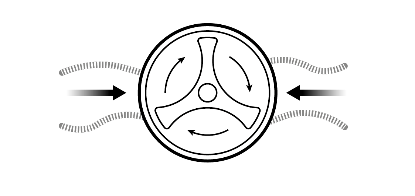 RevoFit2^TM^ | 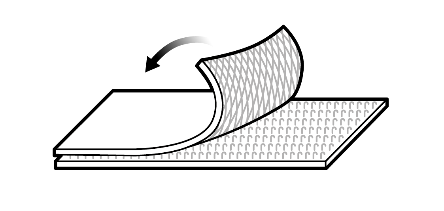Velcro | 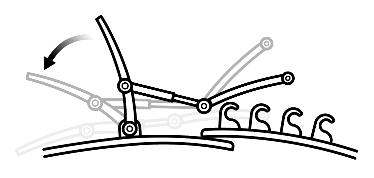Ski boot | - enough force to reliably secure fit  - easy to manipulate and adjust  - not too bulky  - reliable |
| - comfort  - biocompatible  - breathable | inside/cushioning layer | 3MESH spacer fabric | common material (for example: Hapla Fleecy Web) |  | - breathable  - temperature and climate regulation  - high-Quality soft touch  - good formability  - good Pressure distribution  - spring like compressive behaviour  - low weight  - OEKO-TEX Standard 100  (biocompatible) |
| **Requirement** | **Feature** | **Chosen solution** | **Alternative solutions** | **Reasons** | **Requirement** |
| - lightweight  - breathable  - place for electrodes | general shape | holes + bridges | fixed electrode position |  | - breathable  - more air flow  - multiple electrode positions possible |
| - range of motion | joint | 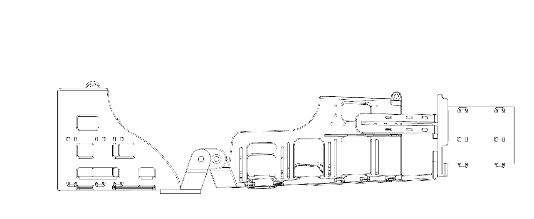double hinge | 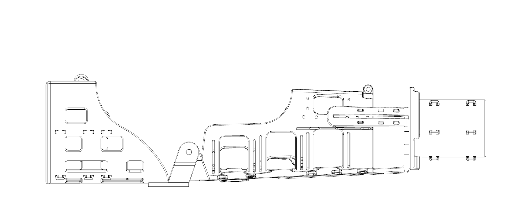single hinge | 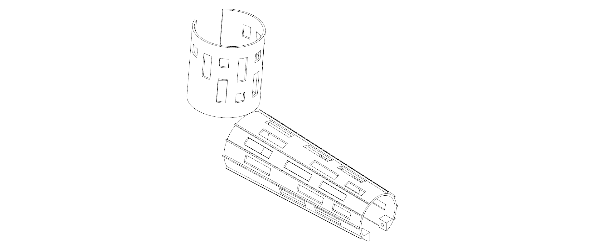loose | - no fixed centre of rotation,  yet real joint/no sliding/stretching of joint |
